# Impact of Different Fecal Processing Methods on Assessments of Bacterial Diversity in the Human Intestine

**DOI:** 10.3389/fmicb.2016.01643

**Published:** 2016-10-20

**Authors:** Yu-Hsin Hsieh, Courtney M. Peterson, Anne Raggio, Michael J. Keenan, Roy J. Martin, Eric Ravussin, Maria L. Marco

**Affiliations:** ^1^Department of Food Science and Technology, University of California, Davis, DavisCA, USA; ^2^Agricultural Biotechnology Center, National Chung Hsing UniversityTaichung, Taiwan; ^3^Pennington Biomedical Research Center, Baton RougeLA, USA; ^4^Louisiana State University Agricultural Center, Baton RougeLA, USA; ^5^Western Human Nutrition Research Center, DavisCA, USA

**Keywords:** 16S rRNA, *Faecalibacterium*, feces/specimen processing method, human intestinal microbiota, fiber, obesity, diet

## Abstract

The intestinal microbiota are integral to understanding the relationships between nutrition and health. Therefore, fecal sampling and processing protocols for metagenomic surveys should be sufficiently robust, accurate, and reliable to identify the microorganisms present. We investigated the use of different fecal preparation methods on the bacterial community structures identified in human stools. Complete stools were collected from six healthy individuals and processed according to the following methods: (i) randomly sampled fresh stool, (ii) fresh stool homogenized in a blender for 2 min, (iii) randomly sampled frozen stool, and (iv) frozen stool homogenized in a blender for 2 min, or (v) homogenized in a pneumatic mixer for either 10, 20, or 30 min. High-throughput DNA sequencing of the 16S rRNA V4 regions of bacterial community DNA extracted from the stools showed that the fecal microbiota remained distinct between individuals, independent of processing method. Moreover, the different stool preparation approaches did not alter intra-individual bacterial diversity. Distinctions were found at the level of individual taxa, however. Stools that were frozen and then homogenized tended to have higher proportions of *Faecalibacterium, Streptococcus*, and *Bifidobacterium* and decreased quantities of *Oscillospira, Bacteroides*, and *Parabacteroides* compared to stools that were collected in small quantities and not mixed prior to DNA extraction. These findings indicate that certain taxa are at particular risk for under or over sampling due to protocol differences. Importantly, homogenization by any method significantly reduced the intra-individual variation in bacteria detected per stool. Our results confirm the robustness of fecal homogenization for microbial analyses and underscore the value of collecting and mixing large stool sample quantities in human nutrition intervention studies.

## Introduction

The intestinal microbiota provide vital contributions to gastrointestinal (GI) and systemic health. The composition of bacterial species and associated metabolites in the human GI tract have been associated with multiple disorders, such as obesity and inflammatory bowel diseases (Ley et al., 2006; Flint et al., 2012; Hooper et al., 2012; Tremaroli and Backhed, 2012; Kabat et al., 2014). Importantly, these bacteria not only serve as indicators for disease but also as targets for therapeutic intervention. Many of the recent developments to our understanding of the intestinal microbiome have been the result of the application of next generation DNA sequencing methods targeting bacterial 16S rRNA genes for phylogenetic and taxonomic assessments of bacterial community membership (Liu et al., 2012). Because of the difficulty in collecting intestinal contents from human subjects, feces are typically used as the source to identify and characterize the intestinal microbiota. Although microbial composition differs along the length of the digestive tract (Eckburg et al., 2005; Stearns et al., 2011), fecal bacteria were shown to have a greater diagnostic value for patients with irritable bowel syndrome than those located in the gut mucosa (Rangel et al., 2015). Therefore, methods used to sample and examine the microorganisms in human fecal material should be optimally designed to ensure precision, accuracy, and a lack of significant bias introduced either by sample collection, homogenization, DNA extraction and sequencing, or data analysis.

Numerous studies have examined the effects of storage conditions, DNA extraction methods, and PCR parameters on the detection and quantification of bacteria in human stools. Several reports concluded that bacterial community composition was not affected by incubating human feces at room temperature for 24 h or 4°C for 48 h or freezing immediately after collection at either -20 or -80°C (Wu G.D. et al., 2010; Cardona et al., 2012; Carroll et al., 2012; Hang et al., 2014; Fouhy et al., 2015; Tedjo et al., 2015). The application of nucleic acid stabilizing reagents (such as RNAlater) have shown promise, but also significant limitations. Although the microbiota of stools stored in RNAlater were stable for 7 days at room temperature (Flores et al., 2015) and were subject-specific over time (Voigt et al., 2015), the incorporation of RNAlater for fecal sample storage decreased DNA quality (Dominianni et al., 2014) and yields (Gorzelak et al., 2015). The microbial diversity (Dominianni et al., 2014) and proportions of certain bacterial taxa were also affected (Nechvatal et al., 2008; Flores et al., 2015; Gorzelak et al., 2015).

Different commercial DNA extraction kits were found to be capable of recovering sufficient quantities of DNA (Scupham et al., 2007; Nechvatal et al., 2008; Salonen et al., 2010; Wu G.D. et al., 2010; Sergeant et al., 2012; Yuan et al., 2012; Hang et al., 2014; Kennedy et al., 2014; Santiago et al., 2014; Vishnivetskaya et al., 2014; Wesolowska-Andersen et al., 2014; Wagner Mackenzie et al., 2015), but yielded proportional changes in identified taxa (Salonen et al., 2010; Wu G.D. et al., 2010; Sergeant et al., 2012; Yuan et al., 2012; Hang et al., 2014; Kennedy et al., 2014; Santiago et al., 2014; Vishnivetskaya et al., 2014; Wesolowska-Andersen et al., 2014). Notably, physical shearing of cells by bead-beating improves DNA recovery (Salonen et al., 2010; Yuan et al., 2012; Santiago et al., 2014; Vishnivetskaya et al., 2014) and generally increases the proportions of Gram-positive bacteria that are difficult to lyse by enzymatic methods (Scupham et al., 2007; Sergeant et al., 2012).

PCR parameters, including the DNA polymerase, oligonucleotides, annealing temperature, and barcoding strategy varied the outcome in bacterial surveys (Frank et al., 2008; Engelbrektson et al., 2010; Wu J.Y. et al., 2010; Sergeant et al., 2012; Brandariz-Fontes et al., 2015; Franzen et al., 2015; Sinclair et al., 2015; Tremblay et al., 2015; Zheng et al., 2015; D’Amore et al., 2016; Derakhshani et al., 2016). The number of PCR amplification cycles altered estimates of low abundance bacterial taxa (Gonzalez et al., 2012) but did not change conclusions of the overall community structure (Wu J.Y. et al., 2010). The impacts of DNA sequencing platforms, including Ion Torrent, 454 pyrosequencing, and Illumina, on the outcomes of assessments of bacterial composition in stool samples were minor (Salipante et al., 2014; Sinclair et al., 2015; Tremblay et al., 2015; D’Amore et al., 2016). In contrast, bioinfomatics approaches can result in significant differences (Bokulich et al., 2013; Majaneva et al., 2015).

Despite these seemingly exhaustive comparisons of microbial analysis techniques, stool preparation methods have not been well-tested. Although the collection of small aliquots of stool is sufficient to perform bacterial analysis (e.g., 200 mg or less), it is not clear how well the bacterial species are distributed throughout the fecal contents. Therefore it is not known whether sampling from different spatial locations in the stool without homogenization is adequate to obtain data that are representative of the stool as a whole. This is particularly relevant for human studies wherein people consumer different foods throughout the day. Moreover, there has not been a comparative analysis of different fecal preparation methods, and less labor-intensive methods have often been favored. In preparation for optimal processing of stool samples from a study of the effects of dietary resistant starch in prediabetic participants, we measured the intra- and inter-individual variations in stool samples with and without freezing and with and without homogenization to evaluate the extent to which the bacterial proportions change as a result of processing steps.

## Materials and Methods

### Sample Collection and Preparation

**FIGURE 1:**
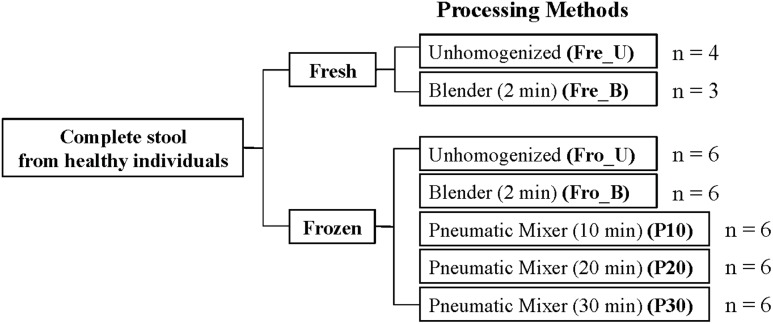
**Fecal sample processing study design.** An “*n*” indicates the number of human subjects for which stools were tested. For each processing method, six technical replicates were performed.

The study design is provided in **Figure 1**. Complete stools were collected from six healthy individuals [Subjects (S) 1–6] and processed within 2 h after collection. A portion of the stool was frozen at -80°C, and the remaining was directly sampled (Fre_U) in <1 g portions or homogenized by blending. For blending, fresh stool was homogenized in a blender (Ninja Master Prep, QB900B, Euro-Pro Operating, Boston, MA) for 2 min (Fre_B). The frozen stool was randomly chipped in <1 g portions (Fro_U), mixed in a blender for 2 min (Fro_B), or homogenized in a pneumatic mixer for either 10, 20, or 30 min (P10, P20, and P30) (Miracle Paint Sport DC-1-C, Minneapolis, MN, USA). For each of those methods, the stools were processed while frozen. Sterile distilled water (4°C) was added to stools in a proportion of 1.5 times the sample weight (63.7 ± 29.9 g) for homogenization in the blender and pneumatic mixer. For homogenization in the pneumatic mixer, the frozen stool and 25 stainless steel balls (0.8 cm in diameter) were agitated in 3.79 l containers. A total of six replicates were collected for each of the seven preparation methods for each of the six participants and stored at -80°C until DNA extraction. The parent study [STARCH study] was approved by the PBRC’s institutional review board, was registered on clinicaltrials.gov (NCT01708694), and all participants provided written informed consent.

### DNA Extraction

DNA was extracted and purified with either the QIAamp DNA Stool Mini Kit (Qiagen, Hilden, Germany; for samples from S1 to S3) or QIAamp Fast DNA Stool Mini Kit (for samples from S4 to S6) according to kit availability and with the following modifications: A total of 200–250 mg processed stool was added to tubes containing 300 mg of 0.1 mm zirconium beads (BioSpec Products, Inc. Bartlesville, OK, USA) suspended in 100 μl lysis buffer (200 mM of NaCl, 100 mM of Tris-HCl, 20 mM of EDTA and 20 mg/ml of lysozyme). After 30 min incubation with 500 rpm mixing at 37°C, the ASL buffer from the QIAamp DNA stool mini kit (or InhibitEX buffer from QIAamp Fast DNA Stool Mini Kit) was added into the sample mixture for homogenization with two cycles on a MP FastPrep-24 instrument (MP Bio MP Biomedicals LLC, Santa Ana, CA, USA) at 6.5 m/s for 1 min. The supernatant was further processed using QIAamp kits according to the manufacturer’s instructions and the purified DNA was stored at -20°C until PCR amplification.

### Library Preparation for Illumina Sequencing

The V4 region of the 16S rRNA gene was amplified using primers 515F and 806R (Caporaso et al., 2011). An eight-nucleotide unique barcode was appended onto the 5′ end of the forward primer for sample identification as previously described (Yin et al., 2014). The target was amplified in a 50 μl reaction containing 5 ng of template DNA, 0.8 U Ex Taq DNA polymerase (Takara Bio Inc. Kyoto, Japan), 1× Ex Taq buffer, 2.5 mM MgCl_2_, 0.1 μM of each primer, and 200 μM dNTP mixture. Thermocycling reactions were as follows: 3 min of denaturation at 94°C, followed by 35 cycles (S1–S3) or 30 cycles (S4–S6) of 45 s at 94°C, 60 s at 50°C and 40 s at 72°C, and with a final extension for 10 min at 72°C. A total of 5 μl of each PCR product was pooled and visualized on an agarose gel prior to purification with a Wizard SV Gel and PCR Clean-Up System (Promega, Fitchburg, WI, USA). The concentration of the purified replicon mixture was measured on a NanoDrop (Thermo Scientific, Waltham, MA, USA) and then used for DNA library preparation and paired-end Illumina Mi-Seq (PE250) sequencing (Illumina Inc., San Diego, CA, USA) at the UC Davis Genome Center^1^.

### 16S rRNA Gene Sequence Analysis

Sequence analysis was performed using the pipeline QIIME version 1.8.0 (Caporaso et al., 2010). DNA sequences with an error in the barcode or those that did not pass a minimum average quality score of 30 were excluded while demultiplexing (-e 0 and -s 30). This resulted in an average of 67,056 ± 11,041 reads per sample, each with an average length of 222 ± 0.3 bases. The sequences were then clustered into OTUs by the open reference OTU picking approach in QIIME with default settings using the UCLUST method (Edgar, 2010) based on 97% sequence similarity against the Greengenes reference database version 13_8 (DeSantis et al., 2006). Upon removing OTUs constituting less than 0.005% of the total observed counts, a total of 935 OTUs in all stool samples remained. For alpha and beta diversity analysis, the samples were rarefied to 10,000 reads. The alpha diversity indices including observed OTUs, PD whole tree, and Shannon diversity were determined. Beta diversity was assessed by the weighted UniFrac distance (Lozupone et al., 2011) and Bray-Curtis dissimilarity, and visualized using PCoA. For alpha diversity and the within processing method distance, raw sequencing data were divided into six groups based on person and analyzed following the process as described above. Raw sequences are available in the NCBI SRA under BoProject ID PRJNA295801.

### Statistical Analysis

Differences in alpha diversity of observed OTUs, PD whole tree, and Shannon indices were assessed by the Kruskal–Wallis test followed by Dunn’s *post hoc* test with a default Bonferroni correction in Graphpad Prism 6 (GraphPad Software Inc., La Jolla, CA, USA). The differences in the relative abundance of bacterial taxa were compared with the Mann–Whitney *U*-test or Kruskal–Wallis test followed by FDR correction in R “stats” package (R 3.1.1). The Dunn’s *post hoc* test in R “dunn.test” package was used to specify the processing method where the taxa were significantly altered. A *P* value < 0.05 was considered significant.

## Results

### Inter-individual Variation in Fecal Bacterial Composition Is Not Altered by Processing Method

**FIGURE 2:**
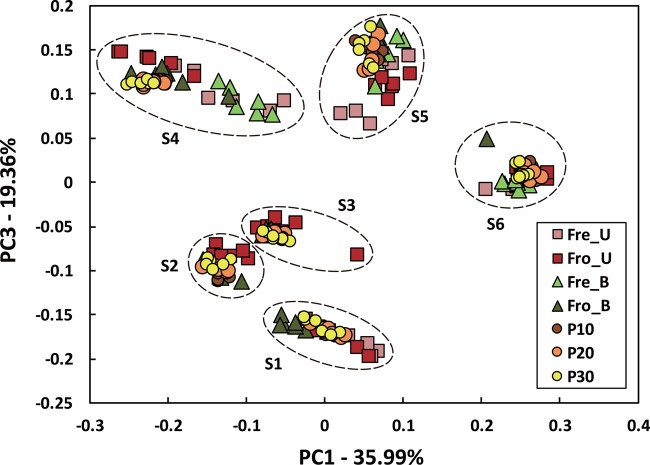
**Inter- and intra-individual variation in microbiota composition according to different fecal processing methods.** Bacterial diversity was visualized by PCoA using the weighted UniFrac metric and colored according to processing method. An “S” indicates subject number.

**FIGURE 3:**
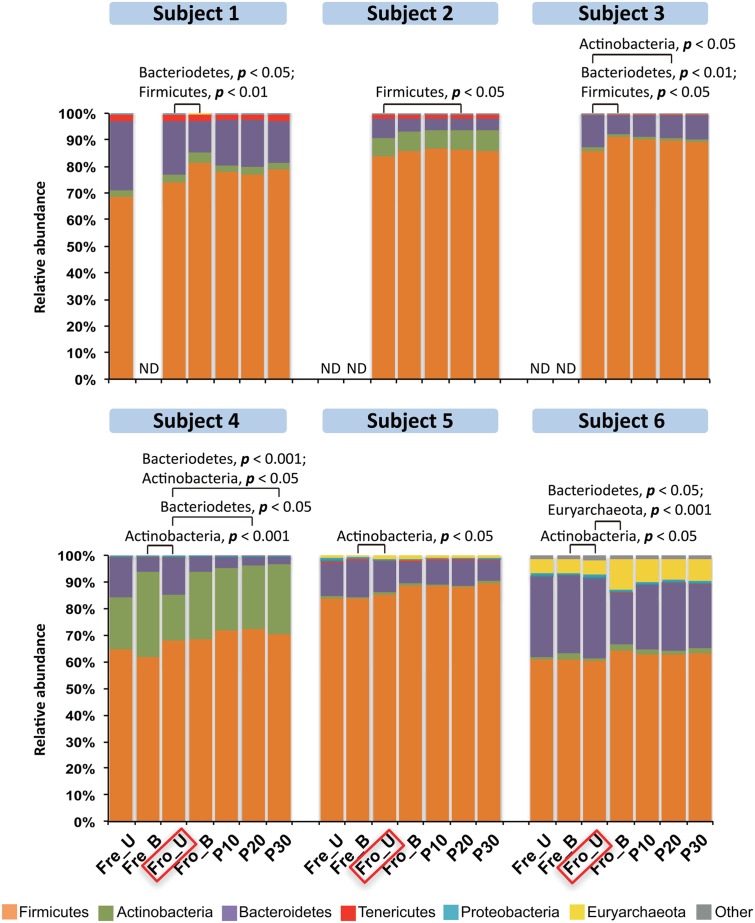
**Phylum-level distributions among the fecal microbiota in six human subjects.** Bars represent the mean abundance of each phylum (*n* = 6 replicates per person per stool sample). Phyla constituting <2% of all sequences are designated as “Other” and, overall, this category constituted less than 4% of the bacteria detected. Frozen un-homogenized samples were used as the reference for paired comparison (indicated in the red box). Significant differences were identified by Mann–Whitney *U*-test. ND, the microbiota was not measured with that processing method.

**FIGURE 4:**
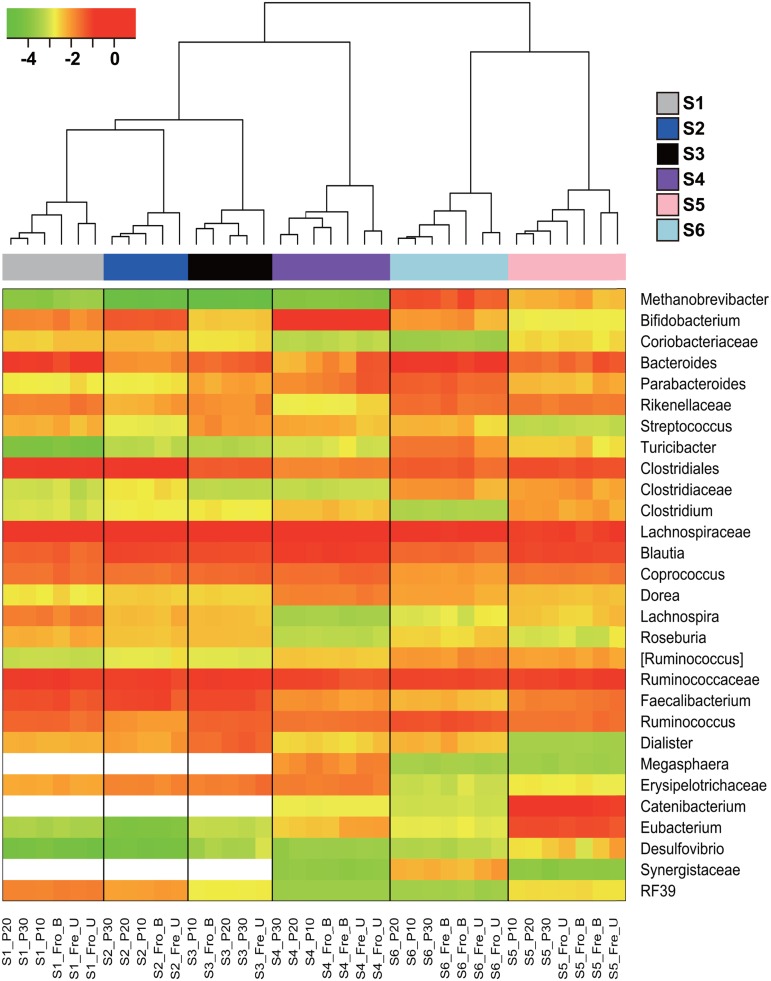
**Hierarchical clustering of the dominant bacterial taxa in fecal samples.** The heat map shows the log_10_-transformed relative abundance of 29 bacterial taxa. Taxa were excluded if they constituted less than 1% in all fecal samples. [Ruminococcus] indicates the OTUs assigned as *Ruminococcus* belonging to the *Lachnospiraceae* family. The *x*-axis contains the subject sample designation including the individuals (S1–S6) and processing methods. The blank cells indicate taxa that were below the detection limit.

Stools from six individuals were collected and prepared as described in **Figure 1**. PCR amplicons of bacterial community 16S rRNA gene V4 regions were subjected to high-throughput DNA sequencing and analysis. Comparisons of all stool preparation methods showed that the six individuals each harbored a distinct fecal microbial profile according to the weighted Unifrac metric (**Figure 2**). Although the different fecal preparation methods did result in some alterations to the bacterial diversity among the individuals, the dispersive level was inconsistent for each person (**Figure 2**). Bray–Curtis dissimilarity metrics confirmed that that subject was the prominent factor for sample clustering (data not shown). Taxonomic comparisons showed the inter-individual differences among the stools (**Figure 3**). At the phylum level, individuals were predominantly populated by *Firmicutes* and with variable levels of *Bacteroides* and *Actinobacteria* (**Figure 3**). Hierarchical clustering of the log-transformed proportions of the most abundant taxa confirmed that the bacterial composition in the fecal samples grouped according to subject and not by processing method (**Figure 4**).

### Intra-individual Variation in Fecal Bacterial Composition is Inconsistently Altered by Processing Method

According to alpha-diversity metrics (number of observed OTUs, PD, and Shannon index), there were no significant differences in bacterial diversity between unhomogenized fresh and frozen feces for any of the six individuals (Supporting Information Table S1). Blending of either frozen or fresh stool also did not result in significant intra-individual alterations to species richness, with the exception of S5 for which there was a significant reduction in PD for the blended fresh (*P* = 0.037) and frozen (*P* = 0.025) fecal material compared to the Fro_U stool samples and reductions for fresh (*P* = 0.002) and frozen (*P* = 0.002) blended stools according to the Shannon index for S2 and S4, respectively (Supporting Information Table S1). The observed OTUs and PD were also similar between fecal samples with the application of the pneumatic mixer for different lengths of time (Supporting Information Table S1). Changes were found for three individuals, however, significance was limited to one or two of the alpha-diversity metrics, no single homogenization time was consistently affected nor was species richness repeatedly increased or decreased with mixing.

Because the fecal sample preparation methods did not result in consistent global changes in bacterial diversity, we next examined for impacts of those methods on the relative abundance of individual taxa. For each subject, bacterial proportions in stool samples that were homogenized tended to be more similar to each other than samples that were not homogenized (**Figure 4**). Consistent with these differences was the general increase in proportions of *Fimicutes* and *Actinobacteria*, phyla-containing Gram-positive bacteria, for frozen stools that were homogenized (**Figure 3**). Although the ratios of *Firmicutes* to *Bacteroidetes* increased for some stools after homogenization, the change was not consistent (**Figure 3**).

**FIGURE 5:**
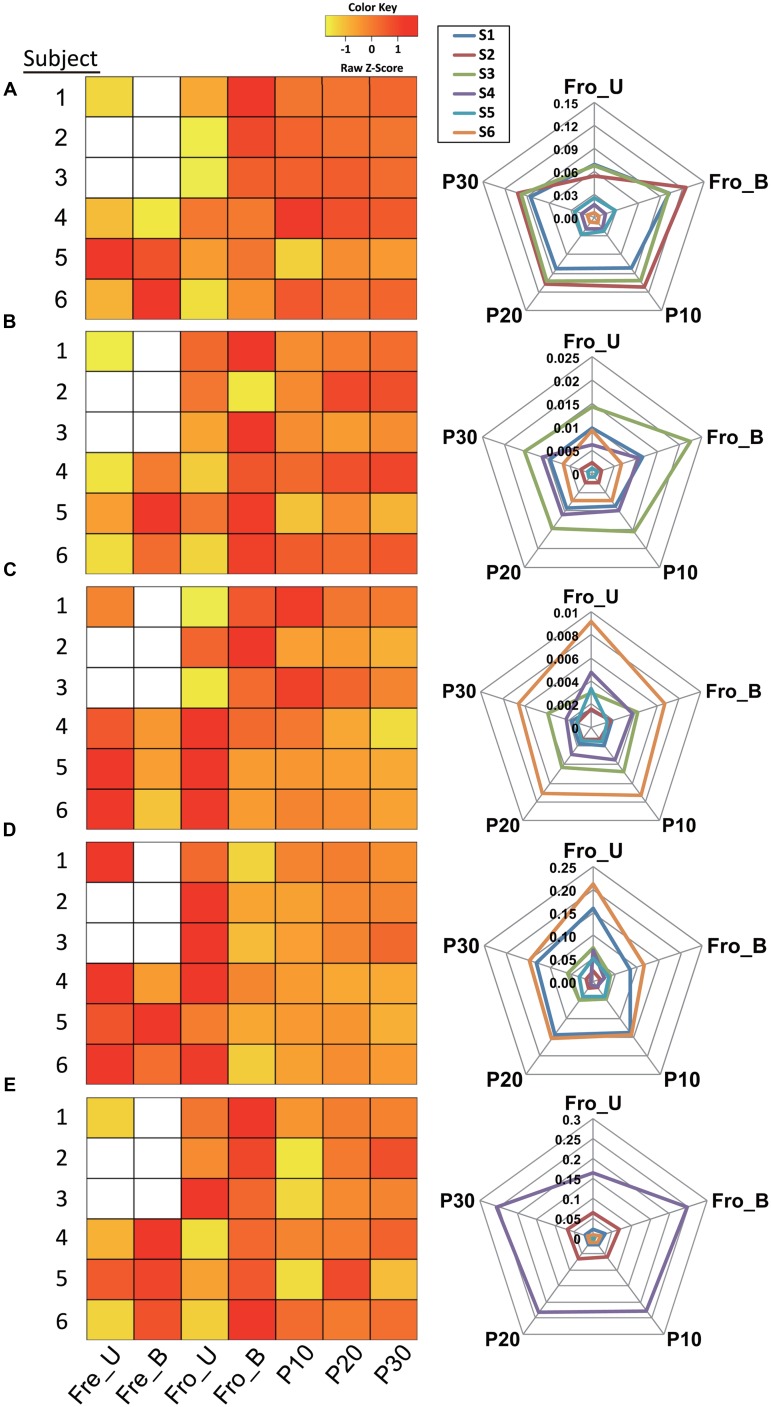
**Fecal processing method effects on bacterial taxa.** Proportional changes of the genera **(A)** *Faecalibacterium*, **(B)** *Streptococcus*, **(C)** *Oscillospira*, **(D)** *Bacteroides*, and **(E)** *Bifidobacterium* depending on processing method. The relative abundances in heat maps were scaled by *z*-score. Blank cells indicate stool samples for which data is not available. The radar charts show the relative abundances for five processing methods.

At deeper taxonomic levels, certain taxa were consistently and reproducibly altered within individual stools depending on freezing or homogenization. Among the 40 identified bacterial families, intra-individual proportions of *Ruminococcaceae* were significantly changed for all individuals, indicating that this family might be susceptible to processing bias (Supporting Information Table S2). In particular, the relative abundance of the genus *Faecalibacterium*, a member of the *Ruminococcaceae* family (*Firmicutes* phylum), was significantly modified by at least one processing method for all of the stools tested (Supporting Information Table S2). Pairwise comparisons of *Faecalibacterium* relative abundances for each subject showed that there was no single processing method that consistently resulted in changes to the proportions of this genus. However, homogenization in general tended to increase the proportions of *Faecalibacterium*, maximally up to 2.1-fold, compared to the unhomogenized samples for each individual (**Figure 5A**; Supporting Information Table S3). Importantly, these results were consistent even with the application of two different DNA extraction methods and PCR amplification conditions (data not shown).

The proportions of several other genera and their corresponding families were also affected by processing method for stools from the majority (≥4) of human subjects. Within the *Firmicutes* phylum, *Streptococcus* (family *Streptococcaceae*), *Oscillospira* (family *Ruminococcaceae*), and SMB53 (candidate genus; family *Clostridiaceae*) were significantly affected by one or more processing methods at both the family and genus levels (Supporting Information Table S2). The SMB53 genus has yet to be defined and therefore is not discussed further here. For five out of six subjects, the proportions of *Streptococcus* increased after the stools were homogenized by blender (**Figure 5B**; Supporting Information Table S3). Conversely, the pneumatic mixer resulted in a lower relative abundance of *Oscillospira* for stools from four out of six subjects (**Figure 5C**; Supporting Information Table S3).

For the *Bacteroidetes* phylum, proportions of *Bacteroides* (*Bacteroidaceae* family) and *Parabacteroides* (*Porphyromona daceae* family) were modified by processing method (Supporting Information Table S2). *Bacteroides* proportions were between 0.2- and 0.78-fold lower following homogenization of frozen stool (by blending or pneumatic mixer) for the majority of subjects (**Figure 5D**; Supporting Information Table S3). Similar trends were found for *Parabacteroides* (data not shown).

Lastly, quantities of *Bifidobacterium*, a member of the *Actinobacteria* phylum, were modified. The proportions of *Bifidobacterium* and effects of processing method varied among subjects. However, the levels of this genus tended to increase when the stools were processed in the blender (**Figure 5E**; Supporting Information Table S3).

### Homogenization-Reduced Intra-sample Variability

**FIGURE 6:**
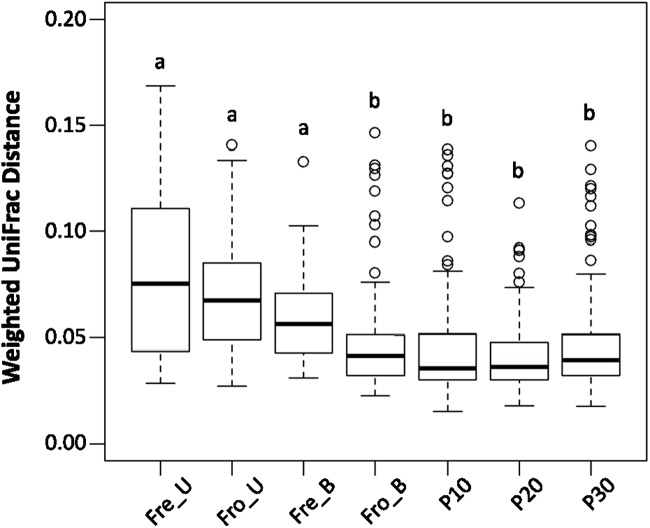
**Homogenization decreased sample variation among replicates.** Fecal samples were grouped according to processing method for calculation of the within group weighted UniFrac distances for each individual. Boxes that do not share a same letter are significantly different (*p* < 0.05) according to the Kruskal–Wallis test with the Dunn’s *post hoc* test.

The within-group, weighted UniFrac distances of the homogenized fecal samples were significantly lower than found for stools that were not homogenized prior to DNA extraction (**Figure 6**). These results show that homogenization by either blending or the pneumatic mixer improves the consistency of the results between technical replicates. Moreover, freezing the stools also influenced the results because the within-group UniFrac distances of fecal samples that were frozen prior to blending (Fro_B) were significantly lower than those that were “fresh” and homogenized shortly after collection (Fre_B) (*P* < 0.0001 by the Mann–Whitney *U*-test; **Figure 6**).

## Discussion

We found that the inter-individual variation in fecal microbiota between subjects was clearly greater than any difference introduced either by freezing or homogenization. Intra-individual comparisons showed that there was no method that reproducibly altered the alpha and beta diversity of the bacteria in the same way among stools from all subjects. Instead, our findings strongly indicate that there are certain families and genera that are more vulnerable to over/under representation in stools based on the processing method and hence might be more susceptible to potential biases caused by the manner in which fecal samples are collected, stored, and processed.

Our findings are in agreement with other studies showing that current protocols for multiplexed, 16S rRNA gene sequence assessments of fecal bacterial diversity are sufficiently robust that inter-individual variation in fecal microbiota is not overcome despite large differences in stool preparation and DNA sequencing and analysis methods (Salonen et al., 2010; Wu G.D. et al., 2010; Carroll et al., 2012; Dominianni et al., 2014; Hang et al., 2014; Santiago et al., 2014; Wesolowska-Andersen et al., 2014; Fouhy et al., 2015; Tedjo et al., 2015; Voigt et al., 2015; Wagner Mackenzie et al., 2015). Importantly, biases introduced by sample preparation and analysis methods are minor compared to the large changes in intestinal bacterial composition caused by diet, antibiotics, and disease (Willing et al., 2011; Perez-Cobas et al., 2013; David et al., 2014; Conlon and Bird, 2015; Arora and Bäckhed, 2016; Matijasic et al., 2016). The greater impact of these biologically, as opposed to technically, induced differences shows that human fecal bacteria can be monitored for species diversity and richness, even with procedural distinctions.

Consistent with this premise, the relative abundances of the dominant bacterial phyla were not consistently affected by freezing or homogenization. Although the proportions of Gram-positive bacteria and the corresponding *Firmicutes* to *Bacteroidetes* ratios tended to increase following homogenization (either by blending or pneumatic mixer), no phylum was repeatedly, significantly enriched (or depleted).

At other taxonomic levels, we found that processing methods can have modest effects on study outcomes. By testing for repeated, significant differences that could be attributed to bacterial families and specific genera within those families, we identified six genera susceptible to variation in stools from the majority (≥4) of the six human subjects tested. Most notably, *Faecalibacterium* was the only microbial taxon for which the relative abundance was significantly affected by one or more processing method in all individuals. Homogenization tended to increase the proportions of this genus, although no single processing method was responsible for these effects. *Faecalibacterium* is a butyrate-producing genus in the *Firmicutes* phylum that has been associated with good intestinal health (Miquel et al., 2013). Members of this genus are anti-inflammatory and secrete proteins that reduce activation of NF-κB pathways in rodent and cell culture models (Quevrain et al., 2016). These bacteria are negatively associated with inflammatory bowel diseases and other inflammatory conditions in humans (Sokol et al., 2008; Eppinga et al., 2016; Matijasic et al., 2016). *Faecalibacterium* was also found in lower proportions in individuals with Type 2 diabetes (T2D; Tilg and Moschen, 2014) and increased in a diet that promoted insulin sensitivity in obese subjects (Haro et al., 2016b).

Similar to *Faecalibacterium*, the levels of *Streptococcus*, another genus in the *Firmicutes* phylum, were increased in stools after homogenization. *Streptococcus* is commonly used in the production of fermented foods (Veiga et al., 2014) and is a prominent member of the human small intestine microbiota (Zoetendal et al., 2012). It was also recently reported that proportions of this genus are reduced in stools of individuals with T2D on a macrobiotic diet (Candela et al., 2016). Notably, because the homogenization methods applied here lowered the levels of *Oscillospira*, another genus associated with human health (Konikoff and Gophna, 2016), it should not be expected that homogenization will increase all physiologically relevant members of the *Firmicutes* phylum.

Other bacteria of medical and ecological significance were also consistently altered by homogenization such that they were either more (*Bifidobacterium*) or less (*Bacteroides* and *Parabacteroides*) abundant. *Bifidobacterium* is commonly applied as a probiotic and is an indigenous inhabitant of the large intestine of both infants and adults (Bottacini et al., 2014; Jost et al., 2015). *Bacteroides* and *Parabacteroides* are also prominent in the intestine and appear to be pivotal, diet-responsive members of the human microbiota (Wu et al., 2011; Del Chierico et al., 2016; Haro et al., 2016a). Taken together, these findings show how the fecal processing method could influence conclusions on the impacts of dietary interventions on bacterial genera with functions important for human health.

Presently, it remains to be shown whether these significantly changed taxa are either more or less vulnerable to homogenization or if there are other factors that influenced the outcomes. One possibility is that certain bacteria are more sensitive to lysis and DNA shearing/degradation and, therefore, were lost with the application of homogenization for sample processing. This effect would lower the detectable numbers of those sensitive taxa and, conversely, increase the proportions taxa able to withstand the sample processing method. Alternatively, homogenization could provide benefits by dislodging bacteria from macromolecules or aggregates and distributing them for increased access during DNA extraction. This change would enable more accurate and reliable detection of those taxa that would otherwise be missed by less rigorous processing approaches. The latter possibility is supported by our finding that homogenization of frozen stools, either by blender or pneumatic mixer, resulted in significant reductions in intra-sample variation. These findings are in agreement with homogenized fecal samples that were examined either by quantitative PCR (Gorzelak et al., 2015) or shot-gun metagenomics (Wesolowska-Andersen et al., 2014). Notably, the outcomes were not solely due to freezing because of the lack of change in bacterial diversity between the fresh and frozen unhomogenized samples.

In summary, fecal sample homogenization is a useful method for characterizing the human intestinal microbiome and reducing intra-sample variation. When at all possible, this method should be used instead of random sampling without homogenization because of the within-stool variation in microbial composition. Moreover, the intra-sample variability was similar for homogenization by blender versus pneumatic mixer. This result is robust evidence both are valid methods for homogenizing stool in research protocols. Because the blender method took only 2 min, in comparison to the more labor-intensive shaker-and-beads method (10–30 min), blending offers a time-saving approach to homogenization. Ultimately, improved understanding of fecal microbiota will be gained from the application of constructed bacterial community “standards” and improved knowledge on the localization of different bacterial taxa at micro- and macro-scales within luminal contents. Because of the sensitivity of certain taxa to be enriched or diminished as a result of fecal processing, comparisons across studies should be viewed with caution and instead more general outcomes provided.

## Author Contributions

Y-HH performed experiments, analyzed data and wrote the manuscript; CP designed the experiments and revised the manuscript; AR performed experiments; MK, RM, and ER designed the experiments and revised the manuscript; MM designed the experiments and wrote the manuscript.

## Conflict of Interest Statement

The authors declare that the research was conducted in the absence of any commercial or financial relationships that could be construed as a potential conflict of interest.

## Footnotes

1 http://dnatech.genomecenter.ucdavis.edu/

## Abbreviations

FDR: false discovery rate
Fre_B: fresh blender
Fre_U: fresh unhomogenized
Fro_B: frozen blender
Fro_U: frozen unhomogenized
OTUs: operational taxonomic units
P10: pneumatic mixer for 10 min
P20: pneumatic mixer for 20 min
P30: pneumatic mixer for 30 min
PCoA: principal coordinates analysis
PD: phylogenetic diversity
QIIME: quantitative insights into microbial ecology
SRA: Sequence Read Archive
