# Supplementary material for: Impact of Different Fecal Processing Methods on Assessments of Bacterial Diversity in the Human Intestine
Source: Front Microbiol. 2016 Oct 20;7:1643. doi: 10.3389/fmicb.2016.01643 (PMC5071325; doi:10.3389/fmicb.2016.01643)
Supplement: Supplementary file 1 [file Table_1.PDF]

Supporting Information Table S1. Alpha diversity of the fecal microbiota for each subject and processing method.

		S1		S2		S3		S4		S5		S6	
	**Diversity indeces**	**Value**	***P***	**Value**	***P***	**Value**	***P***	**Value**	***P***	**Value**	***P***	**Value**	***P***
**Fre_U**	**Observed OTUs**	698.3 (7.3)	NS	NA	NA	NA	NA	440.6 (17.1)	NS	527.9 (8.6)	NS	497.2 (7.1)	NS
	**PD**	40.9 (0.3)	NS	NA	NA	NA	NA	24.3 (0.4)	NS	28.5 (0.3)	NS	28.6 (0.3)	NS
	**Shannon**	7.25 (0.05)	NS	NA	NA	NA	NA	5.81 (0.13)	NS	6.69 (0.18)	NS	6.38 (0.06)	NS
**Fro_U**	**Observed OTUs**	710.9 (11.0)	-	690.4 (9.1)	-	737.9 (7.6)	-	448.7 (10.5)	-	524.2 (7.2)	-	502.8 (8.5)	-
	**PD**	41.4 (0.3)	-	36.6 (0.3)	-	39.9 (0.3)	-	24.6 (0.3)	-	28.7 (0.3)	-	28.6 (0.3)	-
	**Shannon**	7.31 (0.06)	-	7.34 (0.05)	-	7.10 (0.05)	-	5.98 (0.19)	-	6.52 (0.11)	-	6.43 (0.03)	-
**Fre_B**	**Observed OTUs**	NA	NA	NA	NA	NA	NA	436.8 (9.2)	NS	499.2 (8.6)	NS	500 (9.4)	NS
	**PD**	NA	NA	NA	NA	NA	NA	24.3 (0.3)	NS	28.1 (0.3)	0.037	28.3 (0.3)	NS
	**Shannon**	NA	NA	NA	NA	NA	NA	5.44 (0.25)	0.002	6.49 (0.09)	NS	6.46 (0.06)	NS
**Fro_B**	**Observed OTUs**	707.2 (2.8)	NS	692.1 (4.2)	NS	751.1 (5.6)	NS	449.2 (7.4)	NS	497.6 (10.6)	NS	496.4 (13.5)	NS
	**PD**	41.4 (0.3)	NS	36.9 (0.2)	NS	40.5 (0.2)	NS	24.4 (0.2)	NS	28.1 (0.2)	0.025	28.5 (0.3)	NS
	**Shannon**	7.28 (0.02)	NS	7.15 (0.06)	0.002	7.11 (0.05)	NS	5.66 (0.23)	NS	6.11 (0.11)	0.002	6.39 (0.09)	NS
**P10**	**Observed OTUs**	710.4 (4.3)	NS	694.3 (5.2)	NS	754.5 (6.7)	NS	459 (5.3)	NS	492.4 (27.3)	NS	499.6 (6.0)	NS
	**PD**	41.5 (0.5)	NS	36.7 (0.3)	NS	40.5 (0.2)	NS	24.7 (0.1)	NS	27.6 (1.7)	NS	28.5 (0.2)	NS
	**Shannon**	7.27 (0.02)	NS	7.16 (0.04)	0.002	7.12 (0.05)	NS	5.76 (0.04)	NS	6.21 (0.07)	NS	6.43 (0.05)	NS
**P20**	**Observed OTUs**	712.2 (4.6)	NS	703.4 (11.3)	NS	757.6 (6.7)	0.015	462.2 (5.7)	NS	502.8 (10.2)	NS	498.2 (4.1)	NS
	**PD**	41.4 (0.3)	NS	37.1 (0.4)	NS	40.8 (0.2)	< 0.001	24.6 (0.3)	NS	28.3 (0.3)	NS	28.3 (0.3)	NS
	**Shannon**	7.27 (0.02)	NS	7.21 (0.07)	NS	7.10 (0.05)	NS	5.73 (0.07)	NS	6.22 (0.16)	NS	6.42 (0.02)	NS
**P30**	**Observed OTUs**	714.3 (6.7)	NS	697.1 (8.2)	NS	752.7 (5.3)	NS	458.3 (9.8)	NS	490.9 (25.4)	NS	500.1 (6.9)	NS
	**PD**	41.6 (0.4)	NS	36.9 (0.4)	NS	40.5 (0.4)	NS	24.5 (0.3)	NS	27.6 (1.6)	NS	28.5 (0.2)	NS
	**Shannon**	7.28 (0.02)	NS	7.18 (0.)07	NS	7.10 (0.03)	NS	5.65 (0.08)	0.002	6.13 (0.16)	NS	6.41 (0.03)	NS

Frozen unhomogenized (Fro_U) samples were used as the reference for intra-individual comparison to other homogenization methods. Each value represents mean (stdev). P = p-value according to the Mann–Whitney U test. NA: not-available. NS: not-significant.
