# Supplementary material for: Impact of Different Fecal Processing Methods on Assessments of Bacterial Diversity in the Human Intestine
Source: Front Microbiol. 2016 Oct 20;7:1643. doi: 10.3389/fmicb.2016.01643 (PMC5071325; doi:10.3389/fmicb.2016.01643)
Supplement: Supplementary file 2 [file Table_2.PDF]

Supporting Information Table S2. Intra-individual variation in bacterial taxa due to stool processing methods[a]

Phylum	Family	Genus	S1	S2	S3	S 4	S5	S6
Euryarchaeota	*Methanobacteriaceae*	-	0.126[b]	0.973	0.736	0.835	0.056	< 0.001
		Methanobrevibacter	0.167	0.971	0.739	0.792	0.062	< 0.001
Actinobacteria	*Actinomycetaceae*	-	0.844	0.184	0.938	0.004	0.235	0.003
		Actinomyces	0.829	0.156	0.970	0.008	0.320	0.378
		Varibaculum	NA	NA	NA	0.474	0.586	0.002
	Bifidobacteriaceae	-	0.005	0.712	0.018	0.001	0.397	< 0.001
		Bifidobacterium	0.005	0.790	0.028	0.002	0.341	< 0.001
	Coriobacteriaceae	-	0.003	0.074	0.020	0.081	0.009	0.071
		Adlercreutzia	0.002	0.790	0.028	NA	0.029	0.060
		Collinsella	0.359	0.651	0.714	0.178	0.071	0.152
		Eggerthella	0.156	NA	NA	0.234	0.317	0.075
Bacteroidetes	*Bacteroidaceae*	-	0.001	0.184	0.015	< 0.001	< 0.001	< 0.001
		Bacteroides	0.002	0.249	0.016	< 0.001	< 0.001	< 0.001
	Porphyromonadaceae	-	0.005	0.702	0.018	< 0.001	0.005	< 0.001
		Parabacteroides	0.006	0.722	0.024	< 0.001	0.007	< 0.001
	Prevotellaceae	-	0.807	0.108	0.146	< 0.001	< 0.001	0.241
		Prevotella	0.778	0.102	0.159	< 0.001	< 0.001	0.661
	Rikenellaceae	-	0.005	0.010	0.035	0.014	0.117	< 0.001
	S247	-	0.065	0.090	0.736	0.003	0.003	0.228
	Barnesiellaceae	-	0.001	0.006	0.009	0.823	0.363	0.717
	Odoribacteraceae	-	0.130	0.973	0.736	0.168	< 0.001	< 0.001
		Butyricimonas	NA	NA	NA	0.278	< 0.001	< 0.001
		Odoribacter	0.227	0.980	0.714	0.262	< 0.001	0.819
	Paraprevotellaceae	-	NA	NA	NA	0.737	0.009	0.454
Firmicutes	*Gemellaceae*	-	0.331	0.323	0.123	0.489	0.097	0.101
	Enterococcaceae	-	0.311	0.153	0.443	NA	NA	0.029
	Streptococcaceae	-	0.001	0.108	0.018	< 0.001	0.062	< 0.001
		Lactococcus	0.600	0.695	0.970	0.039	0.516	< 0.001
		Streptococcus	0.001	0.104	0.024	< 0.001	0.137	< 0.001
	Turicibacteraceae	-	0.332	0.062	0.600	0.010	0.003	< 0.001
		Turicibacter	0.359	0.052	0.642	0.013	0.003	< 0.001
	Christensenellaceae	-	0.130	0.062	0.292	0.228	0.136	< 0.001
		Christensenella	NA	NA	NA	0.107	0.471	0.022
	Clostridiaceae	-	0.005	0.005	0.149	0.121	0.005	< 0.001
		Clostridium	0.035	0.778	0.188	0.107	0.002	0.661
		SMB53[c]	0.013	0.033	0.692	0.033	0.007	0.001
		Other	0.661	0.833	0.642	NA	0.197	0.006
	Eubacteriaceae	-	NA	NA	NA	0.606	0.363	0.001
		Pseudoramibacter	NA	NA	NA	0.725	0.783	0.002
	Lachnospiraceae	-	0.023	0.184	0.053	0.079	0.005	< 0.001
		Anaerostipes	0.008	0.050	0.462	0.788	0.054	0.002
		Blautia	0.002	0.033	0.401	0.153	0.007	< 0.001
		Coprococcus	0.018	0.007	0.016	0.197	0.007	0.143
		Dorea	0.004	0.126	0.329	0.862	0.174	0.001
		Lachnobacterium	0.007	0.722	0.509	0.046	0.303	0.018
		Lachnospira	0.002	0.007	0.285	0.474	0.013	< 0.001
		Roseburia	0.002	0.029	0.509	0.788	0.004	< 0.001
		Ruminococcus	0.378	0.016	0.412	0.370	0.001	< 0.001
		Other	0.054	0.007	0.028	0.137	< 0.001	0.139
	Peptococcaceae	-	0.505	0.006	0.977	0.976	0.336	0.015
	Peptostreptococcaceae	-	NA	NA	NA	0.521	0.002	0.867
	Ruminococcaceae	-	< 0.001	0.006	0.018	< 0.001	0.001	0.016
		Anaerotruncus	NA	NA	0.425	0.092	0.168	0.002
		Faecalibacterium	< 0.001	0.007	0.028	0.027	0.034	< 0.001

		Oscillospira	0.036	0.078	0.028	0.014	0.003	< 0.001
		Ruminococcus	0.004	0.024	0.506	0.390	0.008	0.001
		Other	0.243	0.790	0.092	NA	0.064	0.273
	Veillonellaceae	-	0.158	0.090	0.015	0.050	0.001	0.005
		Acidaminococcus	NA	NA	NA	0.003	0.316	< 0.001
		Dialister	0.166	0.007	0.016	0.278	0.790	0.002
		Megasphaera	NA	NA	NA	0.153	0.649	0.708
		Mitsuokella	NA	NA	NA	0.664	< 0.001	0.187
		Phascolarctobacterium	NA	NA	NA	0.027	< 0.001	0.011
	Mogibacteriaceae	-	0.003	0.702	0.618	0.521	0.003	0.026
	Tissierellaceae	-	NA	NA	NA	0.164	0.029	0.029
		Parvimonas	NA	0.789	NA	0.161	0.029	0.012
		Sedimentibacter	NA	NA	NA	NA	NA	0.017
	Erysipelotrichaceae	-	0.023	0.062	0.003	0.097	< 0.001	0.002
		Catenibacterium	NA	NA	NA	0.637	< 0.001	0.206
		Coprobacillus	0.763	0.813	0.614	0.109	0.109	0.007
		Holdemania	0.746	0.257	0.122	0.825	0.119	0.120
		Eubacterium	0.215	0.790	0.226	< 0.001	0.004	0.008
		cc_115[c]	NA	NA	NA	0.153	0.557	0.001
Proteobacteria	*Alcaligenaceae*	-	0.001	0.702	0.003	0.256	0.279	0.063
		Sutterella	0.001	0.778	0.004	0.255	0.163	0.063
	Desulfovibrionaceae	-	0.029	0.142	0.018	0.061	< 0.001	0.001
		Bilophila	0.066	0.102	0.016	0.140	0.001	0.001
		Desulfovibrio	0.066	0.790	0.024	0.137	< 0.001	0.013
	Enterobacteriaceae	-	0.113	0.034	0.618	< 0.001	0.029	0.004
		Citrobacter	0.328	0.016	0.138	0.862	0.096	0.553
	Pasteurellaceae	-	0.194	0.687	0.443	0.164	0.002	0.244
		Haemophilus	0.218	0.790	0.433	0.161	0.003	0.249
Synergistetes	*Synergistaceae*	-	NA	NA	NA	0.495	0.613	0.101
Verrucomicrobia	*Verrucomicrobiaceae*	-	0.014	0.446	0.736	0.835	0.029	0.009
		Akkermansia	0.013	0.569	0.853	0.883	0.026	0.009

[a] Taxa composition in each stool was altered by the processing method ($P < 0.0001$ according to PERMANOVA for each subject).

[b] Taxa proportions were compared using Kruskal–Wallis test. The P values were adjusted by false discovery rate correction. NA represents the particular taxon was not identified in the sample.

[c] Candidate genus. Although proportions of these taxa were significantly affected by one or more processing method in the majority of subjects, these general are not well-characterized and therefore not discussed further in the text.
