# Supplementary material for: Impact of Different Fecal Processing Methods on Assessments of Bacterial Diversity in the Human Intestine
Source: Front Microbiol. 2016 Oct 20;7:1643. doi: 10.3389/fmicb.2016.01643 (PMC5071325; doi:10.3389/fmicb.2016.01643)
Supplement: Supplementary file 3 [file Table_3.PDF]

Supporting Information Table S3. The relative abundance of selected bacterial taxa

		Fre_O	Fre_B	Fro_U	Fro_B	P10	P20	P30
Faecalibacterium	**S1**	5.91 (0.33)	NA	6.90 (0.83)	10.26 (0.40)	8.17 (0.41)	8.26 (0.35)	8.58 (0.47)
	**S2**	NA	NA	5.38 (1.06)	12.59 (1.19)	11.22 (1.16)	10.71 (0.70)	10.32 (1.27)
	**S3**	NA	NA	6.71 (0.96)	10.24 (0.65)	10.21 (0.40)	10.28 (0.44)	9.89 (0.75)
	**S4**	1.35 (0.43)	1.22 (0.25)	1.60 (0.29)	1.59 (0.24)	1.86 (0.17)	1.76 (0.11)	1.71 (0.15)
	**S5**	3.92 (1.06)	3.32 (0.54)	2.59 (0.49)	2.96 (0.54)	2.12 (0.85)	2.72 (0.53)	2.60 (0.94)
	**S6**	0.66 (0.07)	0.98 (0.09)	0.58 (0.04)	0.73 (0.03)	0.86 (0.07)	0.81 (0.05)	0.83 (0.06)
Streptococcus	**S1**	0.64 (0.09)	NA	0.97 (0.17)	1.15 (0.03)	0.87 (0.06)	0.92 (0.04)	0.96 (0.04)
	**S2**	NA	NA	0.24 (0.03)	0.22 (0.01)	0.24 (0.02)	0.25 (0.02)	0.25 (0.02)
	**S3**	NA	NA	1.42 (0.41)	2.24 (0.23)	1.55 (0.22)	1.46 (0.11)	1.55 (0.10)
	**S4**	0.55 (0.07)	0.92 (0.18)	0.62 (0.12)	1.07 (0.23)	0.99 (0.04)	1.10 (0.16)	1.14 (0.12)
	**S5**	0.10 (0.01)	0.13 (0.02)	0.11 (0.01)	0.13 (0.01)	0.10 (0.05)	0.11 (0.01)	0.10 (0.05)
	**S6**	0.37 (0.08)	0.81 (0.24)	0.40 (0.08)	1.01 (0.22)	0.88 (0.18)	0.82 (0.11)	0.89 (0.25)
Oscillospira	**S1**	0.17 (0.03)	NA	0.14 (0.01)	0.19 (0.01)	0.20 (0.03)	0.18 (0.03)	0.18 (0.02)
	**S2**	NA	NA	0.15 (0.05)	0.18 (0.02)	0.13 (0.01)	0.13 (0.01)	0.13 (0.01)
	**S3**	NA	NA	0.29 (0.10)	0.42 (0.05)	0.48 (0.04)	0.43 (0.04)	0.39 (0.03)
	**S4**	0.40 (0.17)	0.31 (0.09)	0.48 (0.08)	0.37 (0.05)	0.35 (0.05)	0.29 (0.03)	0.23 (0.01)
	**S5**	0.33 (0.13)	0.15 (0.01)	0.34 (0.06)	0.15 (0.05)	0.16 (0.05)	0.15 (0.03)	0.13 (0.04)
	**S6**	0.97 (0.13)	0.59 (0.10)	0.92 (0.11)	0.67 (0.03)	0.73 (0.06)	0.71 (0.07)	0.66 (0.06)
Bacteroides	**S1**	21.18 (1.58)	NA	15.90 (3.60)	8.52 (0.90)	13.85 (0.59)	14.41 (1.92)	13.06 (1.65)
	**S2**	NA	NA	2.41 (1.05)	1.57 (0.21)	1.59 (0.27)	1.74 (0.20)	1.77 (0.30)
	**S3**	NA	NA	7.32 (3.97)	3.89 (0.40)	4.50 (0.41)	5.10 (0.77)	5.80 (1.14)
	**S4**	7.20 (3.48)	1.44 (0.85)	6.80 (1.56)	2.48 (1.04)	1.40 (0.16)	0.81 (0.15)	0.71 (0.13)
	**S5**	6.36 (1.72)	8.54 (1.03)	4.96 (1.03)	3.53 (0.49)	3.99 (1.03)	4.09 (0.37)	3.24 (0.78)
	**S6**	21.57 (1.81)	17.58 (4.19)	21.24 (2.00)	11.67 (3.64)	14.18 (4.00)	15.38 (3.21)	14.63 (4.32)
Bifidobacterium	**S1**	1.63 (0.35)	NA	2.31 (0.62)	3.12 (0.38)	2.05 (0.19)	2.25 (0.23)	2.21 (0.36)
	**S2**	NA	NA	6.42 (1.07)	6.81 (1.80)	6.01 (0.93)	6.52 (1.31)	6.76 (0.89)
	**S3**	NA	NA	0.63 (0.08)	0.59 (0.04)	0.52 (0.03)	0.56 (0.03)	0.57 (0.02)
	**S4**	19.19 (3.09)	31.13 (5.53)	16.28 (6.04)	24.89 (6.25)	22.67 (1.82)	23.25 (1.32)	25.44 (2.06)
	**S5**	0.29 (0.02)	0.30 (0.03)	0.27 (0.03)	0.29 (0.03)	0.25 (0.10)	0.30 (0.03)	0.26 (0.07)
	**S6**	0.74 (0.11)	1.69 (0.60)	0.77 (0.15)	2.01 (0.70)	1.49 (0.30)	1.38 (0.38)	1.46 (0.51)

Each value represents mean (stdev) in %. NA: not-available.
